# Case Report: rectal perforation and Fournier's gangrene after irinotecan in a bevacizumab-exposed, previously irradiated patient

**DOI:** 10.3389/fonc.2026.1942096

**Published:** 2026-09-24

**Authors:** Libo Wu, Xiaoling Wu

**Affiliations:** 1 Department of Internal Medicine, Emeishan Hospital of Traditional Chinese Medicine, Emeishan, Sichuan, China; 2 Department of Anorectal Surgery, Emeishan Hospital of Traditional Chinese Medicine, Emeishan, Sichuan, China

**Keywords:** bevacizumab, case report, colorectal neoplasms, diagnostic delay, Fournier’s gangrene (perineal necrotizing fasciitis), irinotecan, pelvic irradiation

## Abstract

Bevacizumab-associated intestinal perforation and Fournier’s gangrene have been reported separately; however, the potential contribution of residual anti-VEGF exposure prior to subsequent chemotherapy in previously irradiated pelvic tissue remains underrecognized. Radiotherapy causes fibrosis and microvascular injury, bevacizumab impairs healing, and irinotecan-induced diarrhea may exacerbate perforation risk. Deferring DRE, imaging, and surgical consultation in patients with unexplained perineal or buttock pain increases the risk. A 61-year-old man with rectal adenocarcinoma received neoadjuvant chemoradiotherapy followed by total mesorectal excision and ileostomy in March 2024. After adjuvant chemotherapy, surveillance in March 2025 revealed recurrence with pelvic invasion and liver/lung metastases. He received eight cycles of XELOX plus bevacizumab, then bevacizumab maintenance, stopped on 4 March 2026 for rectal hemorrhage. This 22-day interval is shorter than the label-mandated 28-day hold and NCCN-recommended ≥6-week washout for surgery—standards primarily addressing wound healing rather than chemotherapy sequencing. Five days after irinotecan initiation, grade 3 diarrhea developed. He was admitted on 13 April 2026 with diarrhea, left buttock pain; he received empirical ceftazidime plus moxifloxacin. On 14 April, DRE, imaging, and surgical consultation were deferred due to pain. By 18 April, septic shock developed with perineal necrosis and crepitus. CT confirmed Fournier’s gangrene. Emergency debridement with abscess curettage and seton drainage was performed. Fecal diversion was declined. Antibiotics were escalated to meropenem then vancomycin after cultures grew *VSEfm*. He left the ICU but needed twice-daily dressing changes. Two weeks postoperatively, care shifted to palliation, and he died of multiorgan failure. This case raises the hypothesis that, in previously irradiated tissue, residual bevacizumab exposure at the time of subsequent irinotecan initiation—exacerbated by irinotecan-induced diarrhea and pre-existing rectal mucosal damage—may have contributed to rectal perforation and subsequent Fournier’s gangrene. Notably, while bevacizumab and irinotecan are routinely combined, whether extrapolating surgical washout standards (≥6 weeks) to anti-VEGF sequencing before high-mucosal-toxicity chemotherapy warrants prospective investigation. Unexplained perineal or buttock pain in such patients should trigger urgent clinical evaluation, including DRE when feasible, imaging, and early surgical consultation, to prevent diagnostic delay.

## 1 Introduction

Neoadjuvant chemoradiotherapy combined with total mesorectal excision is the standard for locally advanced rectal cancer (1–3). However, pelvic irradiation can induce chronic tissue fibrosis and microvascular damage, reducing repair capacity (4) and creating a vulnerable tissue environment that may be further destabilized by anti-vascular endothelial growth factor (VEGF) therapy.

Bevacizumab improves survival in metastatic colorectal cancer (mCRC), particularly with irinotecan-based chemotherapy (5), but increases the risks of impaired wound repair and gastrointestinal perforation (6–9).

irinotecan-induced diarrhea can sharply increase luminal pressure. In vulnerable patients, this mechanical stress may exacerbate pre-existing anastomotic weakness and precipitate Fournier’s gangrene. We present a case illustrating how the convergence of these therapies, compounded by delayed recognition of atypical symptoms, culminated in a serious complication. This report highlights the need to maintain a high index of suspicion for bowel rupture in high-risk patients who present with marked diarrhea and unexplained perineal or buttock pain.

## 2 Case description

### 2.1 Past medical history

A 61-year-old man was diagnosed with rectal adenocarcinoma by colonoscopy in October 2023 (**Figures 1A–F**). He had no significant comorbidities, no history of drug or food allergies, and an Eastern Cooperative Oncology Group performance status (ECOG PS) of 0. Pelvic magnetic resonance imaging (MRI) revealed a cT3N1 tumor. Tumor staging was assigned according to the American Joint Committee on Cancer (AJCC) TNM system, 8th edition. The lesion extended beyond the muscularis propria by less than 5 mm and invaded the full thickness of the bowel wall without reaching the internal/external sphincter space. Fewer than four suspicious positive lymph nodes were identified adjacent to the right internal iliac vessels. Both the mesorectal fascia and surrounding vessels were negative. From November 2023 to March 2024, he received preoperative chemoradiotherapy (two cycles of XELOX (oxaliplatin 200 mg intravenously (IV) on day 1 plus capecitabine 1.5 g orally twice daily on days 1–14) plus pelvic intensity-modulated radiotherapy (IMRT), 50.4 Gy/28 fractions with concomitant capecitabine). Laparoscopic TME and temporary ileostomy were performed on March 27, 2024; the tumor was located 6 cm from the anal verge. Postoperative pathology showed moderately differentiated adenocarcinoma invading the deep muscularis propria, with negative circumferential and radial margins and no metastases among six pericolonic lymph nodes examined (tumor regression grade (TRG) score 1, ypT2N0M0, stage I, AJCC 8th edition). He subsequently completed two cycles of adjuvant chemotherapy, underwent planned stoma reversal, and entered scheduled surveillance.

**Figure 1:**
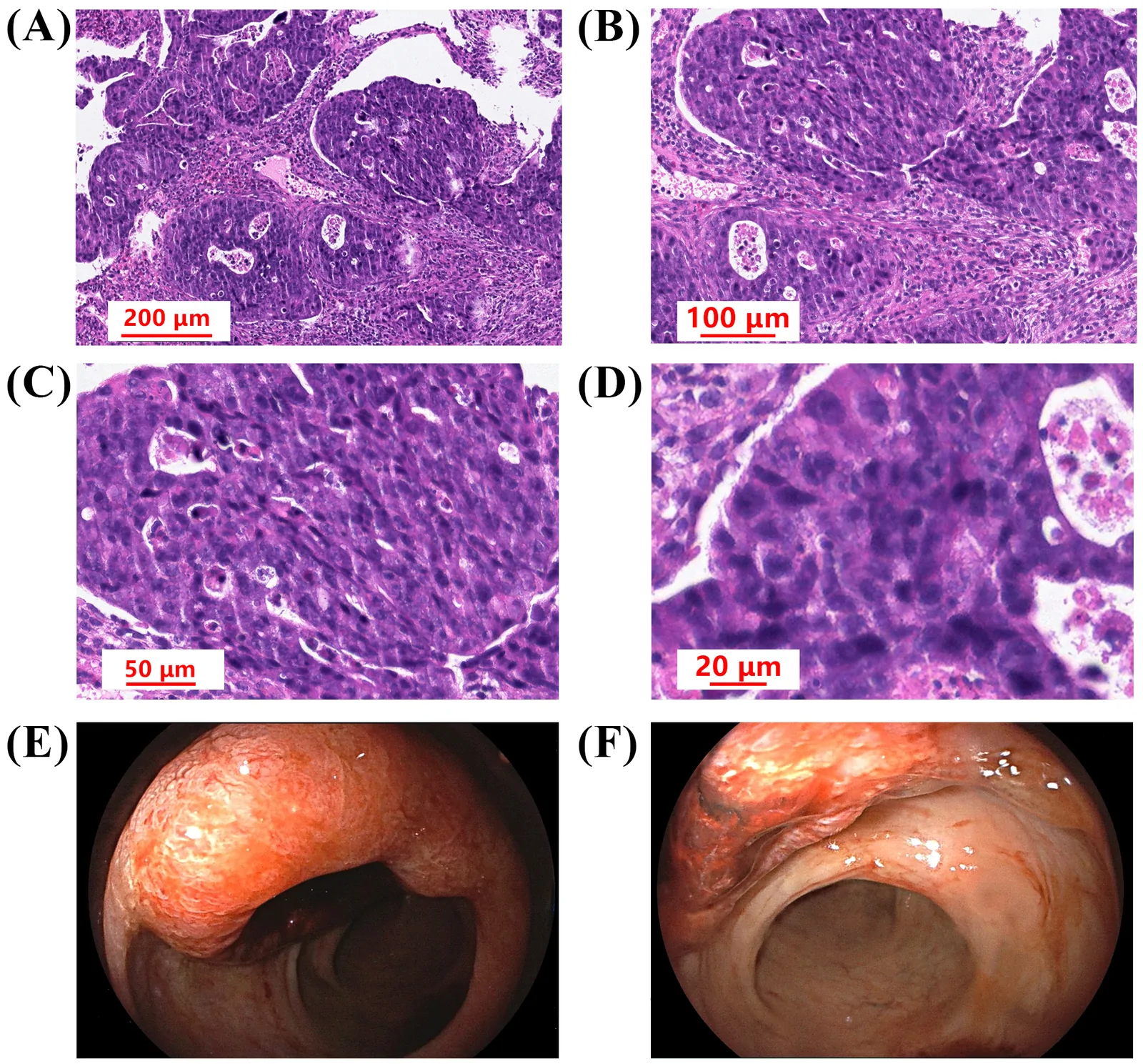
Endoscopic and histological features of the rectal adenocarcinoma specimen. **(A–D)** Representative H&E staining at progressive magnifications (×40–×400) demonstrating irregular glandular architecture, stromal invasion, and marked nuclear pleomorphism. **(E–F)** Representative endoscopic views revealing a protruding, ulcerated mass with mucosal changes, corresponding to the histological sampling site.

### 2.2 Recurrence and systemic therapy

In March 2025, disease progression was confirmed with recurrence at the rectal anastomosis invading the right seminal vesicle, left levator ani, and obturator internus muscles, together with hepatic and pulmonary metastases (rT4bN0M1b, stage IVB, AJCC 8th edition). He received eight cycles of XELOX (oxaliplatin 200 mg IV on day 1 plus capecitabine 1.5 g orally twice daily on days 1–14) plus bevacizumab (600 mg IV on day 1), followed by bevacizumab monotherapy. Bevacizumab was stopped on March 4, 2026, for recurrent rectal hemorrhage—a clinically important adverse event suggesting pre-existing mucosal and vascular damage.

#### 2.2.1 Key clinical course

October 2025 follow-up CT at the referring hospital showed thickening of the rectal anastomosis, perirectal fat stranding, and scattered presacral gas shadows (**Figure 2A**), raising concern for anastomotic leakage or microperforation; the patient was asymptomatic. Nonoperative management with close monitoring was chosen over prompt operative exploration, and bevacizumab was continued until its final administration on 4 March 2026. No documentation was available regarding the specific reasons for this choice. Based on discussions with the patient’s family, it was suggested that the patient may have been reluctant to undergo further surgery at that time, coupled with possible underestimation of anastomotic perforation risk in the setting of prior pelvic irradiation and continued bevacizumab exposure.

**Figure 2:**
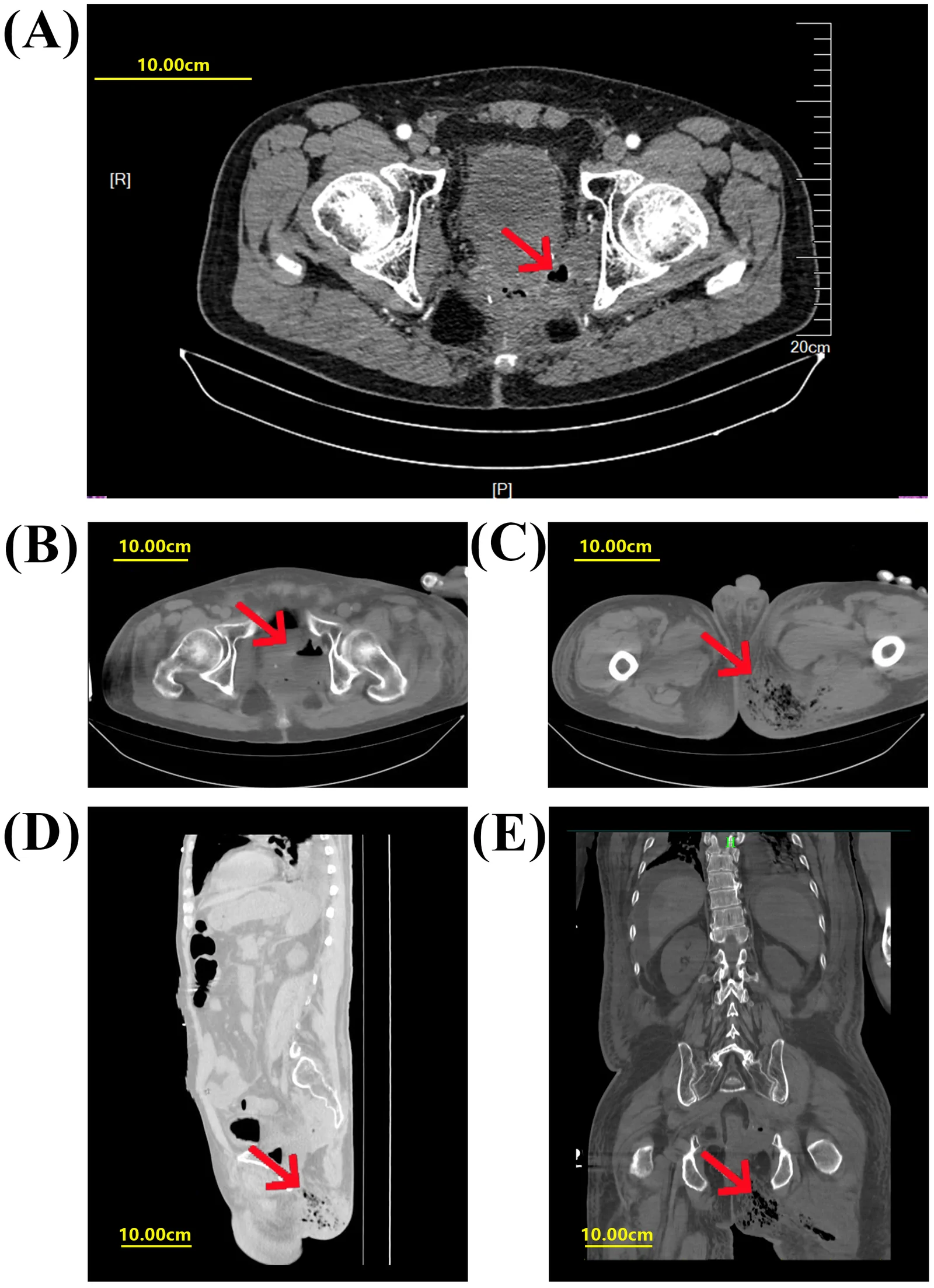
Imaging data of the patient. **(A)** Surveillance CT (Oct 2025) showing rectal anastomotic thickening and presacral gas, suggesting occult perforation. **(B–E)** Emergency CT (Apr 2026) revealing extensive pelvic/perineal necrotizing fasciitis with gas tracking to the left thigh, confirming anastomotic leakage.

### 2.3 Present admission and clinical course

On March 26, 2026, recurrent rectal hemorrhage precluded the original regimen, prompting a switch to S-1 (60 mg twice daily, days 1–14) plus irinotecan (240 mg, day 1) (BSA 2.09 m²; doses reduced given multiple prior lines of chemotherapy and ECOG PS 2). Five days post-infusion, he developed grade 3 diarrhea (CTCAE v5.0: 20–30 watery stools/day) and self-medicated with loperamide and smectite powder without seeking specialist advice. Symptoms worsened, leading to admission on April 13, 2026, with diarrhea, marked left-buttock pain, erythema, swelling, and warmth. Laboratory tests revealed WBC 3.64 × 10^9^/L, CRP 84.09 mg/L, albumin 23 g/L, sodium 126.3 mmol/L, chloride 91.2 mmol/L, and calcium 1.85 mmol/L. Stool examination showed bloody watery stool, occasional phagocytes, leukocytes (3+), erythrocytes (3+), and positive occult blood. Diagnoses included diarrhea, left gluteal soft-tissue infection, hypoalbuminemia, electrolyte disturbance, and postoperative rectal cancer with multiple metastases. Although day-5 diarrhea was consistent with irinotecan-induced late-onset toxicity, the presence of stool erythrocytes and leukocytes, along with severe left gluteal pain, soft-tissue swelling, and systemic inflammation, prompted a proctology consultation to differentiate perianal or deep pelvic abscess from Fournier’s gangrene (necrotizing fasciitis)—an important consideration given that severe, disproportionate pain can be the earliest warning sign of anorectal-origin necrotizing fasciitis. After admission, he received fluid and electrolyte replacement, probiotics, octreotide, antibiotics, and symptomatic care. Empirical ceftazidime plus moxifloxacin was initiated; however, this lacked reliable activity against anaerobes and *Enterococcus* species, which later became clinically important.

#### 2.3.1 Clinical delay

On April 14, a proctology assessment was sought. The digital rectal examination (DRE), cross-sectional imaging, and surgical consultation were deferred because severe left buttock pain limited positioning. This deferral precluded the opportunity to recognize occult perforation at an earlier stage. In high-risk patients (prior pelvic irradiation, recent bevacizumab), new-onset perineal or buttock pain should prompt urgent evaluation, including DRE when feasible, imaging, and early surgical consultation; timely execution of these steps could have enabled earlier perforation recognition. When severe pain precludes an adequate examination, appropriate analgesia or short-acting sedation should be considered.

### 2.4 Acute deterioration and surgical intervention

On the morning of April 18, 2026, the patient deteriorated rapidly. Physical examination revealed dusky-purple necrotic skin, bullae, and epidermal sloughing over the perianal region, perineum, left gluteal region, and posterior thigh, with extensive subcutaneous crepitus palpable (**Figure 3A**). Urgent DRE detected a smooth-walled perforation at the 5 o’clock position, 4 cm proximal to the anal verge, and the gloved finger was coated with foul-smelling pus. Emergent CT confirmed extensive soft-tissue swelling and gas tracking within the left pelvic floor, perineum, and posterior thigh, consistent with necrotizing fasciitis with abscess formation (**Figures 2B–E**). The patient was transferred to the colorectal surgery service for emergency operation.

**Figure 3:**
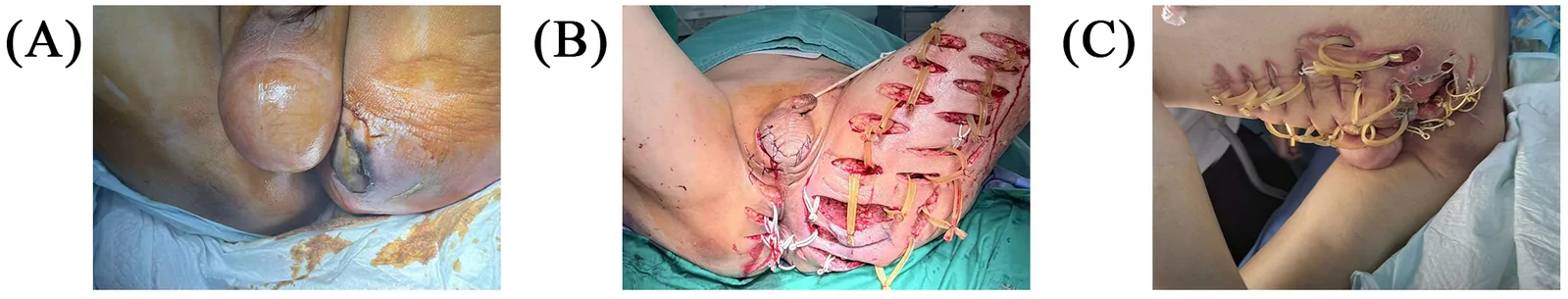
Clinical and surgical images of the patient. **(A)** Preoperative: necrotizing fasciitis of the perineum and left buttock/thigh, showing necrosis, bullae, and subcutaneous crepitus. **(B)** Intraoperative: extensive debridement with passive drain placement. **(C)** Postoperative: massive open wound requiring twice-daily dressing changes.

#### 2.4.1 Operative management

On the afternoon of April 18, 2026, the patient underwent radical debridement of the necrotizing fasciitis, curettage of the perianal abscess cavity, and seton drainage under general anesthesia (**Figure 3B**). Exploration revealed extensive necrosis of the perianal region, perineum, left buttock, and posterior aspect of the left thigh, involving the skin, subcutaneous fat, and superficial and deep fascia. A rectal perforation was identified at the 5 o’clock position, approximately 4 cm from the anal verge.

The surgical procedure included: Debridement extended 1.5–2 cm from the necrotic margin into healthy, viable tissue with fresh bleeding; all potential abscess cavities and fascial planes were fully opened, and necrotic debris and purulent secretions were completely removed. The sinus tract extended from the abscess cavity at the 6 o’clock position to the anal sinus, and a tight rubber-band seton was placed through the internal opening for drainage. The wound was repeatedly irrigated with povidone-iodine solution and normal saline. Rubber drain tubes were placed at the rectal perforation site, perianal debridement wounds, superficial and deep fascial planes, left buttock, and upper left thigh, and were securely fixed to ensure patent drainage without dead angles. The debridement wounds were left open, packed with sterile gauze for drainage. Purulent exudate was sent for bacterial culture to guide postoperative antibiotic therapy.

On April 20, the multidisciplinary team (MDT) concluded that fecal diversion should be deferred due to severe infection, poor nutritional status, and hemodynamic instability. Postoperatively, the wound required twice-daily dressing changes with continuous irrigation and debridement.

#### 2.4.2 Postoperative intensive care

The patient was transferred from the operating room to the intensive care unit (ICU). Persistent hypotension (mean arterial pressure below 65 mmHg), mechanical ventilation without spontaneous breathing, and failure to awaken from anesthesia were noted; lactate was 4.16 mmol/L, WBC 28.08 × 10^9^/L, and CRP 109.65 mg/L. Combined with gluteal infection, these findings indicated septic shock, prompting aggressive treatment. Antibiotics were escalated to meropenem 1 g IV q8h. Initial fluid resuscitation consisted of 2,500 mL crystalloids over 3 hours (29.4 mL/kg for an 85-kg patient). Vasopressor support was initiated with metaraminol 1.18 μg/kg/min (titrated to blood pressure) until 22:00 on April 18, then transitioned to norepinephrine 0.19 μg/kg/min until hemodynamic recovery. Treatment was effective: blood pressure was maintained, vital signs stabilized, consciousness returned, and lactate decreased to 1.89 mmol/L. No acute kidney injury, coagulopathy, or oliguria occurred during the ICU stay.

#### 2.4.3 Microbiology and pharmacotherapy

Wound cultures ultimately grew vancomycin-susceptible *Enterococcus faecium* (*VSEfm*), with resistance to ampicillin, penicillin, erythromycin, and rifampicin; intermediate susceptibility to doxycycline; and susceptibility to vancomycin and linezolid. Concurrent anaerobic culture yielded no obligate anaerobes. Notably, blood cultures were not obtained during the initial resuscitation, precluding bacteremia assessment and representing a limitation of the microbiological evaluation. Guided by susceptibility testing, vancomycin 1 g IV q12h was added on April 22 and maintained until treatment was withdrawn on May 14. Together with source control, this antimicrobial strategy achieved hemodynamic stabilization and transfer out of the ICU.

### 2.5 Outcome

Following ICU discharge, the patient faced a prolonged recovery complicated by a large open operative wound requiring painful, twice-daily dressing changes (**Figure 3C**), with continuous irrigation and debridement of purulent exudate and necrotic debris. Fecal diversion was declined after shared decision-making involving his legally authorized representative and the MDT, given the poor prognosis of his advanced malignancy. Care was progressively redirected toward comfort-focused palliative care measures 2 weeks postoperatively, and the patient eventually died of multiple organ failure.

A temporal summary of the clinical trajectory is provided in **Table 1**.

**Table 1:** Timeline of key clinical events. All data were extracted from the patient’s electronic clinical record.

Date	Episode	Key clinical details
Oct 2023	Identification & Neoadjuvant	Rectal adenocarcinoma; neoadjuvant chemoradiotherapy (pelvic IMRT 50.4 Gy/28 fractions + concurrent capecitabine and oxaliplatin).
Mar 2024	Operation	Laparoscopic TME and temporary ileostomy.
Mar 2025	Recurrence	Pelvic, hepatic, and pulmonary metastases confirmed.
Mar 2025 – Mar 2026	Systemic therapy	Managed with XELOX plus bevacizumab; later switched to single-agent bevacizumab maintenance.
Oct 2025	Follow-up imaging abnormality	CT demonstrated presacral gas shadows; no immediate intervention was performed.
Mar 4, 2026	Final bevacizumab dose	Final dose administered on 4 March 2026; irinotecan initiated 22 days later on 26 March 2026.
Mar 26, 2026	Treatment switch	Switched to S-1 (60 mg) plus irinotecan (240 mg).
Mar 31, 2026	Toxicity onset	Grade 3 diarrhea (CTCAE v5.0); patient self-medicated with loperamide and smectite without specialist consultation.
Apr 9–14, 2026	Diagnostic delay	Intense buttock pain; urgent evaluation, including DRE, imaging, and surgical consultation, was deferred.
Apr 13, 2026	Hospital Admission	Initial empiric ceftazidime+moxifloxacin (inadequate for *Enterococcus/anaerobes*); septic physiology evident.
Apr 18, 2026	Deterioration & Surgery	CT demonstrated gas formation; emergency debridement performed; Septic shock.
Apr – May 2026	ICU care	Sepsis controlled; ongoing wound management required.
Early-Mid May 2026	Outcome	care transitioned to palliative measures; patient died of multiorgan failure.

## 3 Discussion

Necrotizing fasciitis (NF) is a rapidly progressive, life-threatening soft-tissue infection characterized by extensive fascial necrosis (10), and commonly causes sepsis and multiorgan failure, with reported mortality rates of 25%-35% (10, 11). Although most NF cases arise from cutaneous trauma, colorectal rupture is a rare but lethal route of infection; in Fournier’s gangrene, anorectal etiologies account for ~21% of cases and carry the worst prognosis (12).

Although NF secondary to colorectal malignancy has been reported, rapid progression to Fournier’s gangrene triggered by chemotherapy-induced diarrhea in a patient with previous chemoradiotherapy and recent bevacizumab use remains exceptionally rare. This case illustrates a convergence of multiple risk factors in which irradiated tissue fragility, anti-VEGF-mediated impairment of repair, drug-related toxicity and malnutrition-induced immune compromise may have contributed to severe anastomotic failure.

### 3.1 Radiation-induced tissue vulnerability

First, anatomic vulnerability resulted from both local tumor progression and prior irradiation. Recurrent tumor invaded the anastomosis, seminal vesicles, and levator ani muscles, creating a structurally compromised zone prone to breakdown. The chronic phase of radiation injury is characterized by obliterative endarteritis and progressive fibrosis, which markedly reduce tissue oxygen tension and repair potential (4, 13), providing a permissive environment for fulminant soft-tissue infection. Several publications have described Fournier’s gangrene developing during or shortly after neoadjuvant radiotherapy for rectal cancer (14, 15). In our patient, the 50.4 Gy dose administered in 2023 likely contributed to a chronically vulnerable pelvic floor and created the conditions for the subsequent infectious cascade.

### 3.2 Residual antiangiogenic effect of bevacizumab

Second, beyond its anticancer effect, bevacizumab impairs wound healing and is increasingly recognized as a potential precipitant for NF; Gamboa et al. first described Fournier’s gangrene as a possible adverse effect in 2010 (16), and subsequent pharmacovigilance detected 52 cases of necrotizing fasciitis linked to bevacizumab worldwide, including 17 deaths (17). In our patient, bevacizumab had been maintained as monotherapy until stopped for rectal bleeding, suggesting pre-existing mucosal and vascular damage. Furthermore, presacral gas shadows on CT in October 2025 suggest that occult anastomotic perforation may have already been present prior to chemotherapy. Notably, bevacizumab is routinely administered concurrently with irinotecan-based chemotherapy in mCRC, and no pharmacokinetic or pharmacodynamic rationale mandates a washout interval between them (5). However, in the specific context of a previously irradiated pelvis with anastomotic recurrence, irinotecan was initiated 22 days after the last bevacizumab dose. Given bevacizumab’s terminal half-life of ~20 days, substantial exposure likely still persisted at the time of chemotherapy initiation (18); however, the exact magnitude of residual VEGF-inhibitory activity cannot be quantified from half-life alone, as pharmacokinetic persistence does not equate linearly to pharmacodynamic activity, and no pharmacokinetic or pharmacodynamic measurements were performed in this patient. The label-mandated 28-day hold and NCCN-recommended washout of ≥6 weeks pertain primarily to major surgery and wound healing (19), rather than to subsequent cytotoxic chemotherapy. We therefore hypothesize—without implying proven causality—that residual antiangiogenic activity, if present within this interval, may have contributed to anastomotic failure in an already vulnerable, previously irradiated anastomosis; this possibility, and its implications for chemotherapy sequencing, warrant prospective investigation.

### 3.3 Mechanical failure precipitated by irinotecan

Third, irinotecan is mainly associated with myelosuppression and diarrhea, and NF during irinotecan-based treatment for colorectal cancer has rarely been documented (20–22). In this case, grade 3 diarrhea (20–30 episodes/day) generated substantial intraluminal pressure. In the setting of a previously irradiated and bevacizumab-exposed anastomosis, this mechanical stress likely triggered rupture at the site of prior radiological concern. Notably, the October 2025 CT had already shown anastomotic thickening, perirectal inflammatory changes, and presacral gas, raising concern for pre-existing leakage or microperforation. Therefore, severe chemotherapy-associated diarrhea may have aggravated an already structurally compromised anastomosis rather than being the sole cause of a new perforation.

### 3.4 Immune vulnerability related to malnutrition

Fourth, severe diarrhea resulted in the loss of fluids and electrolytes, while gastrointestinal dysfunction led to malabsorption. The patient’s marked hypoalbuminemia (23 g/L) reflected a catabolic state that can impair neutrophil function and gut-barrier defense, thereby facilitating translocation of opportunistic pathogens such as *E. faecium*.

### 3.5 Conclusion of mechanisms

Therefore, this case illustrates a convergence of multiple risk factors rather than a single etiology: a chronically vulnerable pelvic floor following pelvic irradiation, anastomotic recurrence with occult microperforation, residual bevacizumab exposure, irinotecan-induced diarrhea, and malnutrition-related immune compromise. Consequently, whether residual antiangiogenic activity may contribute to anastomotic vulnerability in patients with prior pelvic irradiation and anastomotic recurrence, and whether stringent surgical washout (≥6 weeks) periods should be applied to chemotherapy sequencing remains a hypothesis warranting prospective investigation.

### 3.6 Limitations

This single-case report is limited by the inability to establish definitive causality and the lack of prospective validation. Specifically, bevacizumab serum concentrations were not measured, precluding precise quantification of residual exposure. Multiple competing risk factors, including prior pelvic irradiation, pre-existing anastomotic changes, recurrent tumor invasion, and irinotecan-induced diarrhea, limit causal attribution. Pharmacogenetic information (e.g., UGT1A1 status) was not available. Blood cultures were not obtained, limiting assessment of systemic bacteremia. The effects of diagnostic delay on ultimate outcome are also uncertain.

### 3.7 Reflection on clinical delays

This case featured two diagnostic delays: the decision to continue bevacizumab despite CT signs suggestive of microperforation in October 2025, and the deferral of DRE, imaging, and surgical consultation on April 14 because of pain. Unexplained perineal or buttock pain in patients with prior pelvic irradiation and recent anti-VEGF exposure should trigger urgent evaluation including DRE when feasible, cross-sectional imaging, broad-spectrum antibiotics, and early surgical consultation.

### 3.8 Microbiological insights

The recovery of *Enterococcus faecium*, although vancomycin-susceptible, was clinically significant. The initial empiric protocol of ceftazidime and moxifloxacin lacked reliable activity against *Enterococcus*, because third-generation cephalosporins and fluoroquinolones have intrinsically poor anti-enterococcal coverage according to CLSI M100 (23), and regional surveillance data confirmed moxifloxacin resistance in more than 88% of *E. faecium* isolates (24). This treatment gap emphasizes that immunosuppressed patients with suspected intra-abdominal or perineal sepsis require broad-spectrum, gut-directed coverage, including agents active against vancomycin-susceptible *Enterococcus* and with strong anaerobic activity, consistent with expert guidelines (25). Timely broadening based on susceptibility testing was associated with sepsis control in our case.

### 3.9 Therapeutic dilemma and palliative considerations

Despite successful source control and resolution of sepsis, the patient’s quality of life remained poor owing to the extensive wound and advanced malignancy. Fecal diversion was declined, and care ultimately shifted to palliation; even technically successful surgery may not alter the terminal trajectory in such high-risk presentations.

## 4 Conclusion

In patients with rectal cancer with a history of pelvic irradiation or recent bevacizumab exposure, severe chemotherapy-associated diarrhea should not be dismissed as expected treatment-related toxicity. Clinicians should maintain a low diagnostic threshold for evaluating perineal or buttock pain and should pursue urgent evaluation, including DRE when feasible, cross-sectional imaging, and early surgical consultation. This case further emphasizes that early recognition of occult perforation, timely surgical intervention (e.g., debridement), and culture-directed antimicrobial therapy are important for managing such complex, high-risk presentations. Whether the stringent washout periods mandated for surgery should be applied to anti-VEGF treatment sequencing in patients with prior pelvic irradiation, prior radical pelvic surgery, or intended exposure to high-mucosal-toxicity cytotoxic regimens (e.g., irinotecan-based chemotherapy) remains a hypothesis warranting prospective investigation.

## 5 Patient perspective

From the family’s perspective, the patient’s quality of life deteriorated significantly during the clinical course. The admission for systemic anticancer therapy was complicated by grade 3 diarrhea, followed by the development of perineal necrotizing fasciitis requiring emergency debridement. Despite technical success of source control, the patient faced a prolonged recovery with a large open wound requiring twice-daily dressing changes. Given the poor prognosis of advanced malignancy and the burden of wound management, the patient, through his legally authorized representative, declined fecal diversion and opted for comfort-focused palliative care. The decision to withdraw life-sustaining therapy was made after careful consideration, reflecting the patient’s previously expressed values and the limitations of further aggressive measures in the setting of terminal malignancy.

## Data Availability

The original contributions presented in the study are included in the article/supplementary material, further inquiries can be directed to the corresponding author/s.
